# Negative Body Image Is Not Related to Spontaneous Body-Scaled Motoric Behavior in Undergraduate Women

**DOI:** 10.3389/fpsyg.2019.00580

**Published:** 2019-03-26

**Authors:** Klaske A. Glashouwer, Charlotte Meulman, Peter J. de Jong

**Affiliations:** ^1^ Department of Clinical Psychology and Experimental Psychopathology, University of Groningen, Groningen, Netherlands; ^2^ Department of Eating Disorders, Accare Child and Adolescent Psychiatry, Groningen, Netherlands

**Keywords:** negative body image, body-scaled motoric behavior, body size overestimation, anorexia nervosa, body schema, eating disorders

## Abstract

Body image disturbance is a core characteristic of anorexia nervosa, which might be grounded in distortions in schematic body representations. In line with this, several studies showed that when walking through door-like apertures of different widths, individuals with anorexia nervosa move as if their bodies are larger than they actually are. They turn their body at a higher aperture/shoulder width ratio than healthy individuals. We examined whether oversized body-scaled motoric behaviors may not be restricted to anorexia nervosa but concern a general feature of negative body image attitudes. Therefore, we investigated the relation between negative body image as assessed with shape and weight concerns subscales of the Eating Disorder Examination Questionnaire and aperture/shoulder width turning ratios in women with a healthy weight (*n* = 62). We found that a more negative body image was unrelated to higher aperture/shoulder width turning ratios. Bayes analysis provided moderate evidence for the null hypothesis that spontaneous body-scaled motoric behaviors are not involved in negative body image. Future studies should disentangle whether being underweight per se is related to distinctive spontaneous body-scaled motoric behaviors or whether an “oversized” body schema is a unique characteristic of anorexia nervosa, potentially contributing to the persistence of this disorder.

“A disturbance in the way one’s body weight and shape is experienced” is considered a core characteristic of anorexia nervosa (AN; American Psychiatric Association, 2013). Body image disturbance is typically divided into an attitudinal component comprising negative feelings and cognitions with respect to weight and shape and a perceptual component referring to an overestimation of body size (e.g., Cash and Deagle, 1997; Gardner and Brown, 2014). In addition, body image disturbances are thought to manifest themselves on a behavioral level, in behaviors such as body checking, body avoidance, or dieting (Cash and Pruzinsky, 1990; Cash, 2011). Empirical studies demonstrated that individuals with AN indeed show a disturbance in their body image both at an attitudinal level (e.g., Cash and Deagle, 1997) and at a perceptual level (Gardner and Brown, 2014; Mölbert et al., 2017). Recently, it has been proposed that these false beliefs of individuals with AN about their body size are grounded in distortions of their schematic body representations (Keizer et al., 2013; Gadsby, 2017). Body schema is defined as a central representation of the spatial properties of the body, which is critical for guiding movement. Specifically, it was hypothesized that individuals with AN not only experience themselves as larger/fatter than they are, but that also the schematic representations of their body would be “oversized.” This hypothesis was tested by comparing the bodily rotations of individuals with and without AN while walking through apertures of different widths. Several studies showed that individuals with AN indeed start turning their body at relatively high aperture/shoulder width (A/S) ratios, indicating that they spontaneously move as if their bodies are larger than they actually are (estimation tasks: Guardia et al., 2010, 2012; Metral et al., 2014; actual behavioral tasks: Keizer et al., 2013; Metral et al., 2014).

Such biased inclination to turn at higher A/S ratios could be restricted to AN but may also represent a more general feature of persistent negative body image attitudes that are also apparent in healthy weight individuals with shape and weight concerns. However, a study in undergraduate women and men did not find a relation between weight and shape concern and estimated spatial requirements for passage (Wignall et al., 2017). In addition, a recent study among healthy adult women indicated that body image concerns do not moderate the relation between estimated and actual aperture passing ability (Irvine et al., 2019). Both findings suggest that the accuracy of estimation of passing ability does not differ as a function of body image concerns when individuals are explicitly asked to estimate passing ability. An important next step is to investigate whether similar findings are obtained when using a more implicit behavioral index of aperture passing. Therefore, the current study was designed to measure actual spontaneous body-scaled motoric behavior in a sample of undergraduate women with a healthy weight. We tested the hypothesis that in women with a healthy weight, higher levels of weight and shape concern would be related to rotating the shoulders at wider apertures.

## Method

### Participants

Participants were recruited via the first-year psychology student participant pool of the University of Groningen in which students received course credits in exchange for participation. We selected participants with a healthy self-reported BMI (range 18.5–25) scoring in the highest or lowest quartile on negative body image. One hundred and thirty-six students of 314 students who filled in an online survey were invited and 62 women participated from the Dutch (*n* = 19) and English (*n* = 43) educational psychology program (*M*_age_ = 19.58; *SD* = 1.45, range = 17–24; *M*_BMI laboratory_ = 21.95, *SD* = 2.16, range = 18.65–27.25; see also Table 1). The study was approved by the Ethical Committee Psychology of the University of Groningen (14043). Participants gave written informed consent prior to their participation.^1^

**Table 1:** Means and standard deviations per group.

	Total sample		High negative body image	Low negative body image	Between groups test
	*n* = 62		*n* = 26	*n* = 36	
	*M (SD)*	Median (*IQR*)	*M (SD)*	*M (SD)*	*t (p)*
Age	19.58 (1.45)	19.00 (1.00)	19.08 (0.97)	19.91 (1.63)	−2.24 (0.02)
BMI (measured in the lab)	21.95 (2.16)	21.60 (3.10)	22.78 (1.91)	21.35 (2.16)	2.69 (<0.01)
EDE-Q WSC (measured in the lab)	1.98 (1.62)	1.27 (2.93)	3.70 (0.82)	0.75 (0.59)	16.51 (<0.001)
Critical A/S	1.30 (0.09)	1.30 (0.20)	1.29 (0.10)	1.30 (0.09)	−0.59 (0.55)
Max rotation for A/S = 0.9 (in °)	71.68 (13.10)	73.72 (15.92)	71.30 (9.10)	71.96 (15.48)	−0.19 (0.85)
Max rotation for critical A/S (in °)	19.15 (7.48)	17.00 (10.14)	16.82 (6.08)	20.88 (8.02)	−2.16 (0.04)
Shoulder width (cm)	40.65 (2.14)	40.50 (3.13)	41.42 (2.29)	40.10 (1.88)	2.49 (0.02)

A/S = aperture/shoulder width ratio; BMI = body mass index; EDE-Q WSC = Eating Disorder Examination Questionnaire, Weight and Shape Concern subscales.

### Materials

#### Negative Body Image

The five-item weight concern and eight-item shape concern subscales of the Eating Disorder Examination Questionnaire (EDE-Q WSC; Fairburn and Beglin, 2008) assess participants’ body image over the last 28 days. Specifically, items reflect the affective-evaluative and cognitive-behavioral dimensions of body image, as defined by Cash (2011). The average of both subscales was used to index negative body image (range: 0–6). Subscales showed high internal consistency within this study (*α*s were 0.87 and 0.95).

### Spontaneous Body-Scaled Motoric Behavior

Participants walked through apertures of different widths created by adjusting the distance between two wooden screens *relative to the shoulder width of the participant* (cf. Keizer et al., 2013). Directly above the screens, a camera was set up to record each trial (Warren and Whang, 1987). Trials were conducted with aperture/shoulder width (A/S) ratios from 0.9 to 1.8 in steps of 0.1. Each aperture width was tested twice resulting in a total of 20 trials. Each trial yielded two measurements of shoulder rotation (walking toward the table and back) resulting in 40 rotations for each participant. The order of A/S ratios was random and the same for each participant to optimize conditions for testing individual differences.

As part of a cover story, participants performed a bogus haptic memory task during the aperture task (cf. Keizer et al., 2013). Participants touched two objects in concealed boxes placed on opposite sides of the room and were asked to indicate to what extent the objects were identical. In between participants walked a distance of 620 cm through the apertures. We told the participants that the position of the screens would change after each trial to “increase difficulty of the task.” Only one participant accurately guessed the actual purpose of the task.

### Procedure

The order of assessments/tasks was fixed. Participants’ height, shoulder width, and weight were measured followed by the aperture task. Subsequently, participants completed the EDE-Q. Finally, participants performed an implicit association test that was included for exploratory purposes unrelated to the current study.

### Data Analyses

#### Data Reduction

Video recordings of the area 1 m before/after the aperture were rated still for still by two independent observers to determine the largest rotation angle for each recording. The ratings showed good reliability with a mean intraclass correlation coefficient of 0.89 (*SD* = 0.11). Participant’s critical aperture width (*A/S_crit_*) was defined as the mean A/S ratio at which that participant started rotating her shoulders above a baseline level of rotational body sway. This was determined by plotting participant’s mean angle of rotation against aperture width. By means of visual inspection, the aperture width immediately preceding the curve’s asymptote was selected as the participant’s critical value (Warren and Whang, 1987).

#### Statistical Analyses

We calculated the bivariate non-parametric Kendall’s tau-b correlation between EDE-Q WSC and *A/S_crit_* because *A/S_crit_* only showed five response levels and EDE-Q WSC was not normally distributed. To correct for the potential confounding influence of BMI, we conducted a hierarchical regression analysis on *A/S_crit_* with BMI in step 1 and EDE-Q WSC in step 2 as predictors. To increase confidence in our results, we complemented these analyses with a Bayesian approach using the free software JASP with a default stretched beta prior width of 1 (JASP Team, 2017). *BF_10_* will be reported for significant tests, and *BF_01_* will be reported for non-significant findings. BFs higher than 3 are interpreted as moderate evidence, between 10 and 30 as strong evidence, and above 30 as very strong evidence (Lee and Wagenmakers, 2013).

## Results

### Descriptives

*A/S_crit_* was undetermined for one participant, which was therefore excluded from the analyses. Means of the descriptive variables per group are presented in Table 1. Individuals high in negative body image on average were younger, had a higher BMI and higher shoulder width, and showed a smaller rotation angle for the critical A/S. We explored whether *A/S_crit_* was related to age and shoulder width, but this was not the case (age: *r* = −0.15, *p* = 0.17, *BF_01_* = 1.42; shoulder width: *r* = 0.01, *p* = 0.92, *BF_01_* = 5.97).

### Relation Between Negative Body Image and Spontaneous Body-Scaled Motoric Behavior

**Figure 1:**
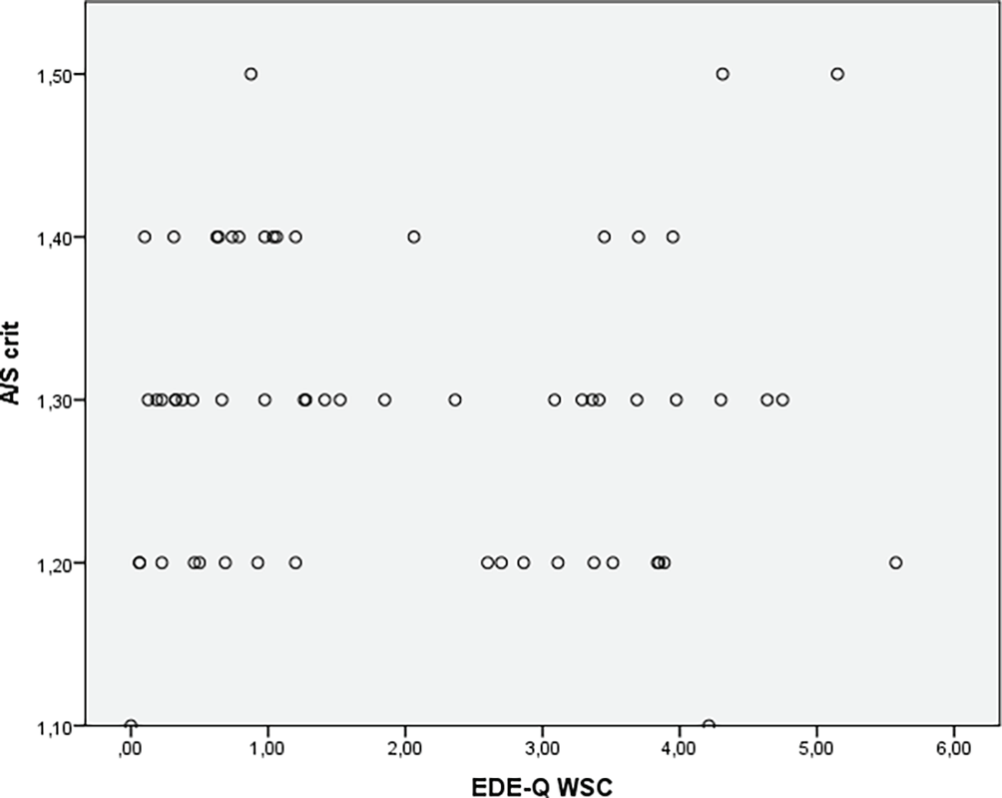
Scatterplot of the correlation between negative body image (EDE-Q WSC) and critical A/S ratio.

Correlational analyses showed that negative body image was unrelated to *A/S_crit_* (*r* = 0.00, *p* = 0.98, *BF_01_* = 6.00; see Supplementary Material for sequential analysis and robustness check; see Figure 1 for scatterplot). BMI was indeed related to negative body image (*r* = 0.26, *p* = < 0.01, *BF_10_* = 14.93), but not to *A/S_crit_* (*r* = −0.07, *p* = 0.51, *BF_01_* = 4.40). Since both variables did not significantly correlate with *A/S_crit_*, we did not conduct the planned regression analysis.

## Discussion

In contrast to our hypothesis, this study showed moderate evidence for negative body image attitudes being *unrelated* to spontaneous body rotation for relatively wide apertures in healthy weight undergraduate women. Thus, different from patients with AN (Keizer et al., 2013; Metral et al., 2014), healthy weight individuals with a negative body image on average did not spontaneously start turning their body for wider apertures than individuals without body image concerns. These findings are in line with two recent studies in healthy adult women in which individuals were explicitly asked to estimate passing ability (Wignall et al., 2017; Irvine et al., 2019). In addition, the results are in accordance with another study showing no difference between individuals with and without obesity with respect to actual turning behavior, even though obese individuals scored significantly higher on body dissatisfaction than the healthy weight group (Scarpina et al., 2017). The average aperture width for which participants in the present study started to turn their body is comparable to prior findings in healthy individuals (*A/S_crit_* = 1.30; cf. Warren and Whang, 1987; Keizer et al., 2013; Scarpina et al., 2017; but see Metral et al., 2014). Together, the empirical findings so far seem to indicate that oversized spontaneous body-scaled motoric behavior (and an oversized body schema cf. Gadsby, 2017) is not generally related to negative body image attitudes but rather is a unique characteristic of AN. If it indeed would play a causal role in the persistence of AN, it would be important to develop specific treatment strategies targeting body schema (e.g., Keizer et al., 2018). However, an alternative explanation would be that all individuals with relatively low BMIs tend to overestimate their size irrespective of their body image (so-called contraction bias; see e.g., Cornelissen et al., 2013; Wignall et al., 2017). In contrast to this idea and to prior findings (Wignall et al., 2017), BMI and shoulder width in the present study were not significantly related to turning behavior. However, having an underweight body *per se* could still be related to distinctive spontaneous body-scaled motoric behaviors. Since the present study did not include individuals with a BMI below the healthy range, an important next step would be to compare spontaneous turning behavior of individuals with AN with underweight individuals without an eating disorder. It would also be informative to use a within-subjects design and examine potential changes in body-scaled action in individuals who change drastically in body size in the short term (e.g., athletes, actors, pregnant women, and pre-post bariatric surgery). In addition, it would be important to examine whether spontaneous body-scaled motoric behavior is oversized in individuals with an eating disorder with a healthy weight, for example, bulimia nervosa, and, whether individuals recovered from AN still show oversized body-scaled behavior.

An important limitation of the current study is that we did not assess the perceptual component of body image disturbance. There are indications that the perceptual component might partially explain the relation between estimated and actual passing ability of an aperture, particularly in individuals with negative body image attitudes (Irvine et al., 2019). Therefore, it could be that the current design did not entirely grasp the complex interplay between all different body image components. In addition, although the present sample size was larger than prior studies in this domain that used behavioral tasks (*n* = 19 per group in Keizer et al., 2013; *n* = 14 per group in Metral et al., 2014; *n* = 18 per group in Scarpina et al., 2017), it should be mentioned that the current sample size was still modest. Therefore, we added a Bayesian statistical approach to determine the amount of evidence for the null hypothesis. Finally, it should be mentioned that the data points in our study might not be entirely independent, since we included measurements of shoulder rotation both from the starting point toward the table as well as from the way back from the table to the starting point.

To conclude, the findings do not support the hypothesis that a heightened tendency to rotate the body for relatively wide apertures is a general feature of individuals with negative body image attitudes. The current failure to find a relationship between negative body image and body rotation in the context of healthy BMI critically differs from previous findings in individuals with AN. Future studies should disentangle whether having an underweight body *per se* is related to distinctive spontaneous body-scaled motoric behavior or whether this is a unique characteristic of AN, potentially contributing to the persistence of this disorder.

## Data Availability

The datasets generated for this study are available on request to the corresponding author. The raw data supporting the conclusions of this manuscript are available through https://dataverse.nl/dataset.xhtml?persistentId=hdl:10411/LDMCT1.

## Author Contributions

All authors contributed to the design of the study. CM and KG conducted the data collection. KG conducted the final processing and data analyses and wrote the first draft of the manuscript. PJ helped to finalizing the manuscript. All authors have approved the final manuscript.

### Conflict of Interest Statement

The authors declare that the research was conducted in the absence of any commercial or financial relationships that could be construed as a potential conflict of interest.

## Footnotes

^1^We originally planned to also include individuals with a relatively low BMI (<18.5) scoring low on negative body image. However, since we only could include a small group (*N* = 12) these data were not included in the present paper.
