# Supplementary material for: Negative Body Image Is Not Related to Spontaneous Body-Scaled Motoric Behavior in Undergraduate Women
Source: Front Psychol. 2019 Mar 26;10:580. doi: 10.3389/fpsyg.2019.00580 (PMC6443978; doi:10.3389/fpsyg.2019.00580)

**Supplementary material**

*Correlation EDE-Q WSC and A/S_crit_*

##### Prior and Posterior


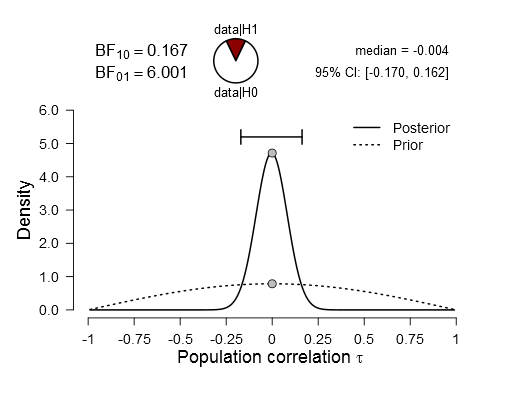


##### Bayes Factor Robustness Check


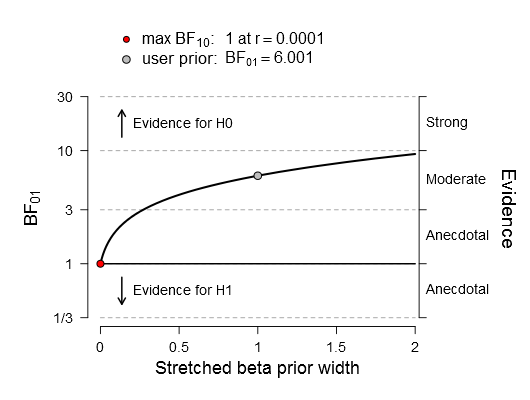


##### Sequential Analysis


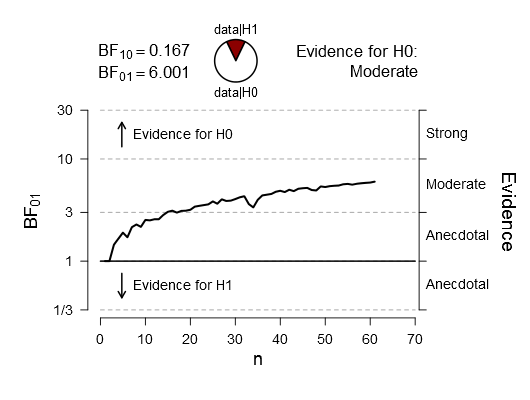


*Correlation BMI and A/S_crit_*

##### Prior and Posterior


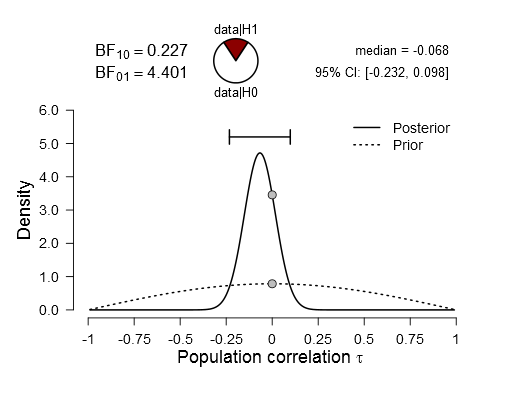


##### Bayes Factor Robustness Check


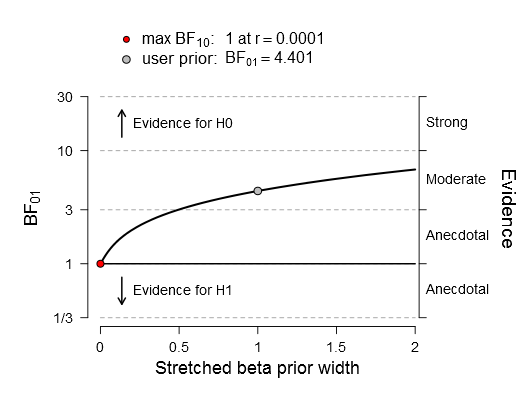


##### Sequential Analysis


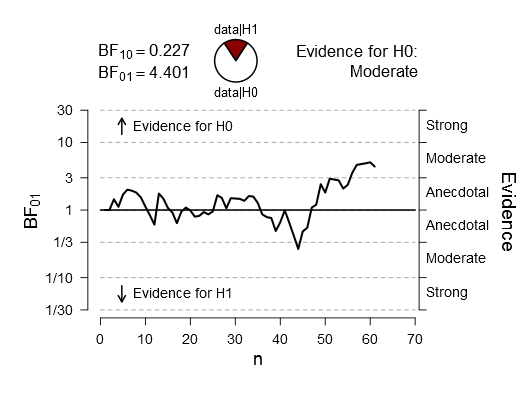

Supplement: Supplementary file 1 [file Table_1.DOCX]
